# Supplementary material for: Uniform genomic data analysis in the NCI Genomic Data Commons
Source: Nat Commun. 2021 Feb 22;12:1226. doi: 10.1038/s41467-021-21254-9 (PMC7900240; doi:10.1038/s41467-021-21254-9)
Supplement: Supplementary file 1 — Supplementary Information [file 41467_2021_21254_MOESM1_ESM.pdf]

Contig_Name	Virus_Name	GenBank_Accession	PaVE_ID	Sequence Revised
CMV	Human Cytomegalovirus, Human herpes virus 5	AY446894.2	N/A	
EBV	Epstein-Barr virus, , Human herpes virus 4	AJ507799.2	N/A	
HBV	Hepatitis B	X04615.1	N/A	
HCV-1	Hepatitis C	AF009606.1	N/A	
HCV-2	Hepatitis C	AF177036.1	N/A	
HIV-1	Human immunodeficiency virus 1	AF033819.3	N/A	
HIV-2	Human immunodeficiency virus 2	M30502.1	N/A	
KSHV	Kaposi's sarcoma-associated herpesvirus, Human herpes virus 8	AF148805.2	N/A	
HTLV-1	Human T-lymphotropic virus 1	AF033817.1	N/A	
MCV	Merkel cell polyomavirus	HM011556.1	N/A	
SV40	Simian vacuolating virus 40	J02400.1	N/A	
HPV16	Human papillomavirus 16	K02718	HPV16REF	Yes
HPV18	Human papillomavirus 18	X05015	HPV18REF	Yes
HPV26	Human papillomavirus 26	X74472	HPV26REF	
HPV31	Human papillomavirus 31	J04353	HPV31REF	
HPV33	Human papillomavirus 33	M12732	HPV33REF	
HPV35	Human papillomavirus 35	X74477	HPV35REF	
HPV39	Human papillomavirus 39	M62849	HPV39REF	
HPV45	Human papillomavirus 45	X74479	HPV45REF	
HPV51	Human papillomavirus 51	M62877	HPV51REF	
HPV52	Human papillomavirus 52	X74481	HPV52REF	
HPV53	Human papillomavirus 53	X74482	HPV53REF	Yes
HPV56	Human papillomavirus 56	X74483	HPV56REF	Yes
HPV58	Human papillomavirus 58	D90400	HPV58REF	
HPV59	Human papillomavirus 59	X77858	HPV59REF	Yes
HPV66	Human papillomavirus 66	U31794	HPV66REF	
HPV68	Human papillomavirus 68	DQ080079	HPV68REF	
HPV69	Human papillomavirus 69	AB027020	HPV69REF	
HPV73	Human papillomavirus 73	X94165	HPV73REF	
HPV82	Human papillomavirus 82	AB027021	HPV82REF	Yes
HPV1	Human papillomavirus 1	V01116	HPV1REF	Yes
HPV2	Human papillomavirus 2	X55964	HPV2REF	
HPV3	Human papillomavirus 3	X74462	HPV3REF	
HPV4	Human papillomavirus 4	X70827	HPV4REF	
HPV5	Human papillomavirus 5	M17463	HPV5REF	Yes
HPV6	Human papillomavirus 6	X00203	HPV6REF	Yes
HPV7	Human papillomavirus 7	X74463	HPV7REF	
HPV8	Human papillomavirus 8	M12737	HPV8REF	
HPV9	Human papillomavirus 9	X74464	HPV9REF	
HPV10	Human papillomavirus 10	X74465	HPV10REF	
HPV11	Human papillomavirus 11	M14119	HPV11REF	
HPV12	Human papillomavirus 12	X74466	HPV12REF	
HPV13	Human papillomavirus 13	X62843	HPV13REF	
HPV14	Human papillomavirus 14	X74467	HPV14REF	Yes
HPV15	Human papillomavirus 15	X74468	HPV15REF	Yes
HPV17	Human papillomavirus 17	X74469	HPV17REF	Yes

HPV19	Human papillomavirus 19	X74470	HPV19REF	
HPV20	Human papillomavirus 20	U31778	HPV20REF	
HPV21	Human papillomavirus 21	U31779	HPV21REF	
HPV22	Human papillomavirus 22	U31780	HPV22REF	
HPV23	Human papillomavirus 23	U31781	HPV23REF	
HPV24	Human papillomavirus 24	U31782	HPV24REF	
HPV25	Human papillomavirus 25	X74471	HPV25REF	
HPV27	Human papillomavirus 27	X74473	HPV27REF	
HPV28	Human papillomavirus 28	U31783	HPV28REF	
HPV29	Human papillomavirus 29	U31784	HPV29REF	
HPV30	Human papillomavirus 30	X74474	HPV30REF	
HPV32	Human papillomavirus 32	X74475	HPV32REF	
HPV34	Human papillomavirus 34	X74476	HPV34REF	
HPV36	Human papillomavirus 36	U31785	HPV36REF	
HPV37	Human papillomavirus 37	U31786	HPV37REF	
HPV38	Human papillomavirus 38	U31787	HPV38REF	
HPV40	Human papillomavirus 40	X74478	HPV40REF	
HPV41	Human papillomavirus 41	X56147	HPV41REF	
HPV42	Human papillomavirus 42	M73236	HPV42REF	
HPV43	Human papillomavirus 43	AJ620205	HPV43REF	
HPV44	Human papillomavirus 44	U31788	HPV44REF	
HPV47	Human papillomavirus 47	M32305	HPV47REF	
HPV48	Human papillomavirus 48	U31789	HPV48REF	
HPV49	Human papillomavirus 49	X74480	HPV49REF	
HPV50	Human papillomavirus 50	U31790	HPV50REF	
HPV54	Human papillomavirus 54	AF436129	HPV54REF	
HPV57	Human papillomavirus 57	X55965	HPV57REF	
HPV60	Human papillomavirus 60	U31792	HPV60REF	
HPV61	Human papillomavirus 61	U31793	HPV61REF	
HPV62	Human papillomavirus 62	AY395706	HPV62REF	
HPV63	Human papillomavirus 63	X70828	HPV63REF	
HPV65	Human papillomavirus 65	X70829	HPV65REF	
HPV67	Human papillomavirus 67	D21208	HPV67REF	
HPV70	Human papillomavirus 70	U21941	HPV70REF	
HPV71	Human papillomavirus 71	AB040456	HPV71REF	Yes
HPV72	Human papillomavirus 72	X94164	HPV72REF	Yes
HPV74	Human papillomavirus 74	AF436130	HPV74REF	
HPV75	Human papillomavirus 75	Y15173	HPV75REF	
HPV76	Human papillomavirus 76	Y15174	HPV76REF	
HPV77	Human papillomavirus 77	Y15175	HPV77REF	
HPV78	Human papillomavirus 78	KC138720	HPV78REF	
HPV80	Human papillomavirus 80	Y15176	HPV80REF	
HPV81	Human papillomavirus 81	AJ620209	HPV81REF	
HPV83	Human papillomavirus 83	AF151983	HPV83REF	
HPV84	Human papillomavirus 84	AF293960	HPV84REF	
HPV85	Human papillomavirus 85	AF131950	HPV85REF	
HPV86	Human papillomavirus 86	AF349909	HPV86REF	
HPV87	Human papillomavirus 87	AJ400628	HPV87REF	Yes
HPV88	Human papillomavirus 88	EF467176	HPV88REF	

HPV89	Human papillomavirus 89	AF436128	HPV89REF	
HPV90	Human papillomavirus 90	AY057438	HPV90REF	
HPV91	Human papillomavirus 91	AF419318	HPV91REF	
HPV92	Human papillomavirus 92	AF531420	HPV92REF	
HPV93	Human papillomavirus 93	AY382778	HPV93REF	
HPV94	Human papillomavirus 94	AJ620211	HPV94REF	
HPV95	Human papillomavirus 95	AJ620210	HPV95REF	
HPV96	Human papillomavirus 96	AY382779	HPV96REF	
HPV97	Human papillomavirus 97	DQ080080	HPV97REF	
HPV98	Human papillomavirus 98	FM955837	HPV98REF	
HPV99	Human papillomavirus 99	FM955838	HPV99REF	
HPV100	Human papillomavirus 100	FM955839	HPV100REF	
HPV101	Human papillomavirus 101	DQ080081	HPV101REF	
HPV102	Human papillomavirus 102	DQ080083	HPV102REF	
HPV103	Human papillomavirus 103	DQ080078	HPV103REF	
HPV104	Human papillomavirus 104	FM955840	HPV104REF	
HPV105	Human papillomavirus 105	FM955841	HPV105REF	
HPV106	Human papillomavirus 106	DQ080082	HPV106REF	
HPV107	Human papillomavirus 107	EF422221	HPV107REF	
HPV108	Human papillomavirus 108	FM212639	HPV108REF	Yes
HPV109	Human papillomavirus 109	EU541441	HPV109REF	
HPV110	Human papillomavirus 110	EU410348	HPV110REF	
HPV111	Human papillomavirus 111	EU410349	HPV111REF	Yes
HPV112	Human papillomavirus 112	EU541442	HPV112REF	
HPV113	Human papillomavirus 113	FM955842	HPV113REF	
HPV114	Human papillomavirus 114	GQ244463	HPV114REF	Yes
HPV115	Human papillomavirus 115	FJ947080	HPV115REF	
HPV116	Human papillomavirus 116	FJ804072	HPV116REF	
HPV117	Human papillomavirus 117	GQ246950	HPV117REF	
HPV118	Human papillomavirus 118	GQ246951	HPV118REF	
HPV119	Human papillomavirus 119	GQ845441	HPV119REF	
HPV120	Human papillomavirus 120	GQ845442	HPV120REF	
HPV121	Human papillomavirus 121	GQ845443	HPV121REF	
HPV122	Human papillomavirus 122	GQ845444	HPV122REF	
HPV123	Human papillomavirus 123	GQ845445	HPV123REF	
HPV124	Human papillomavirus 124	GQ845446	HPV124REF	
HPV125	Human papillomavirus 125	FN547152	HPV125REF	
HPV126	Human papillomavirus 126	AB646346	HPV126REF	
HPV127	Human papillomavirus 127	HM011570	HPV127REF	
HPV128	Human papillomavirus 128	GU225708	HPV128REF	
HPV129	Human papillomavirus 129	GU233853	HPV129REF	
HPV130	Human papillomavirus 130	GU117630	HPV130REF	
HPV131	Human papillomavirus 131	GU117631	HPV131REF	
HPV132	Human papillomavirus 132	GU117632	HPV132REF	
HPV133	Human papillomavirus 133	GU117633	HPV133REF	
HPV134	Human papillomavirus 134	GU117634	HPV134REF	
HPV135	Human papillomavirus 135	HM999987	HPV135REF	
HPV136	Human papillomavirus 136	HM999988	HPV136REF	
HPV137	Human papillomavirus 137	HM999989	HPV137REF	

HPV138	Human papillomavirus 138	HM999990	HPV138REF	
HPV139	Human papillomavirus 139	HM999991	HPV139REF	
HPV140	Human papillomavirus 140	HM999992	HPV140REF	
HPV141	Human papillomavirus 141	HM999993	HPV141REF	
HPV142	Human papillomavirus 142	HM999994	HPV142REF	
HPV143	Human papillomavirus 143	HM999995	HPV143REF	
HPV144	Human papillomavirus 144	HM999996	HPV144REF	
HPV145	Human papillomavirus 145	HM999997	HPV145REF	
HPV146	Human papillomavirus 146	HM999998	HPV146REF	
HPV147	Human papillomavirus 147	HM999999	HPV147REF	
HPV148	Human papillomavirus 148	GU129016	HPV148REF	
HPV149	Human papillomavirus 149	GU117629	HPV149REF	
HPV150	Human papillomavirus 150	FN677755	HPV150REF	
HPV151	Human papillomavirus 151	FN677756	HPV151REF	
HPV152	Human papillomavirus 152	JF304768	HPV152REF	
HPV153	Human papillomavirus 153	JN171845	HPV153REF	
HPV154	Human papillomavirus 154	JN211193	HPV154REF	
HPV155	Human papillomavirus 155	JF906559	HPV155REF	
HPV156	Human papillomavirus 156	JX429973	HPV156REF	
HPV159	Human papillomavirus 159	HE963025	HPV159REF	
HPV160	Human papillomavirus 160	AB745694	HPV160REF	
HPV161	Human papillomavirus 161	JX413109	HPV161REF	
HPV162	Human papillomavirus 162	JX413108	HPV162REF	
HPV163	Human papillomavirus 163	JX413107	HPV163REF	
HPV164	Human papillomavirus 164	JX413106	HPV164REF	
HPV165	Human papillomavirus 165	JX444072	HPV165REF	
HPV166	Human papillomavirus 166	JX413104	HPV166REF	
HPV167	Human papillomavirus 167	KC862318	HPV167REF	
HPV168	Human papillomavirus 168	KC862317	HPV168REF	
HPV169	Human papillomavirus 169	JX413105	HPV169REF	
HPV170	Human papillomavirus 170	JX413110	HPV170REF	
HPV171	Human papillomavirus 171	KF006398	HPV171REF	
HPV172	Human papillomavirus 172	KF006399	HPV172REF	
HPV173	Human papillomavirus 173	KF006400	HPV173REF	
HPV174	Human papillomavirus 174	HF930491	HPV174REF	
HPV175	Human papillomavirus 175	KC108721	HPV175REF	
HPV178	Human papillomavirus 178	KJ130020	HPV178REF	
HPV179	Human papillomavirus 179	HG421739	HPV179REF	
HPV180	Human papillomavirus 180	KC108722	HPV180REF	
HPV184	Human papillomavirus 184	HG530535	HPV184REF	
HPV197	Human papillomavirus 197	KM085343	HPV197REF	
HPV199	Human papillomavirus 199	KJ913662	HPV199REF	
HPV-mCG2	Human papillomavirus -mCG2	JF966378	HPV-mCG2nr	
HPV-mCG3	Human papillomavirus -mCG3	JF966379	HPV-mCG3nr	
HPV-mCH2	Human papillomavirus -mCH2	KF791917	HPV-mCH2nr	
HPV-mFD1	Human papillomavirus -mFD1	JF966375	HPV-mFD1nr	
HPV-mFD2	Human papillomavirus -mFD2	JF966376	HPV-mFD2nr	
HPV-mFS1	Human papillomavirus -mFS1	JF966373	HPV-mFS1nr	
HPV-mFi864	Human papillomavirus -mFi864	KC311731	HPV-mFi864nr	

HPV-mKC5	Human papillomavirus -mKC5	JX444073	HPV-mKC5nr	
HPV-mKN1	Human papillomavirus -mKN1	JF966371	HPV-mKN1nr	
HPV-mKN2	Human papillomavirus -mKN2	JF966372	HPV-mKN2nr	
HPV-mKN3	Human papillomavirus -mKN3	JF966374	HPV-mKN3nr	
HPV-mL55	Human papillomavirus -mL55	KF482069	HPV-mL55nr	
HPV-mRTRX7	Human papillomavirus -mRTRX7	U85660	HPV-mRTRX7nr	
HPV-mSD2	Human papillomavirus -mSD2	KC113191	HPV-mSD2nr	

Supplementary Information Table 1. Virus Sequences included in the GDC Reference Genome.
